# Prevalence, severity, and risk indicators of gingival inflammation in a multi-center study on South American adults: a cross sectional study

**DOI:** 10.1590/1678-775720160178

**Published:** 2016

**Authors:** Paola Carvajal, Mariel Gómez, Sabrina Gomes, Ricardo Costa, Andres Toledo, Fernando Solanes, Hugo Romanelli, Rui Oppermann, Cassiano Rösing, Jorge Gamonal

**Affiliations:** 1 Universidade de Chile, Facultad de Odontología, Departamento de Odontología Conservadora, Laboratorio de Biología Periodontal, Santiago, Chile; 2 Universidad Maimónides, Facultad de Odontología, Buenos Aires, Argentina; 3 Universidade Federal do Rio Grande do Sul, Faculdade de Odontologia, Departamento de Odontologia Conservadora, Porto Alegre, Brasil

**Keywords:** Gingival diseases, Periodontal index, South America

## Abstract

**Objectives:**

The aim of this study is to investigate the prevalence and severity of gingival inflammation and associated risk indicators in South American adults.

**Material and Methods:**

Multi-stage samples totaling 1,650 adults from Porto Alegre (Brazil), Tucumán (Argentina), and Santiago (Chile) were assessed. The sampling procedure consisted of a 4-stage process. Examinations were performed in mobile dental units by calibrated examiners. A multivariable logistic regression model was utilized for associating variables as indicators of gingival inflammation (GI) (Gingival Index ≥0.5). Statistical significance was set at 0.05.

**Results:**

A total of 96.5% of the adults have GI. Regarding the severity of GI, 22.5% of participants examined have mild GI, 74.0% have moderate GI, and 3.6% have severe GI. The multivariate analyses identify the main risk indicators for GI as adults with higher mean of Calculus Index (OR=18.59); with a Visible Plaque Index ≥30% (OR=14.56); living in Santiago (OR=7.17); having ≤12 years of schooling (OR=2.18), and females (OR=1.93).

**Conclusions:**

This study shows a high prevalence and severity of gingival inflammation, being the first one performed in adult populations in three cities of South America.

## INTRODUCTION

Gingival inflammation (GI) is a common clinical feature detected in children and adults^1^. It is characterized by swelling, redness, and bleeding at the gums and it is described as an inflammatory reaction upon the pro-inflammatory cytokines that modulate the balance between humoral and cell-associated immune responses^28^. This clinical feature is characteristic of both gingivitis and periodontitis. GI is considered to be one major class of periodontal conditions, and is recognized to result from the increase in supragingival plaque and the ensuing interactions between the microbiota of biofilm and host response^26^. Consequently, the prevention of plaque accumulation and early treatment of GI reduce the risks associated with the development of the more destructive periodontal disease^5^, which has also been associated with systemic conditions^8^.

Understanding the epidemiologic pattern of GI is essential for planning appropriate public-health services. It has been clearly demonstrated that plaque-induced GI is prevalent at all ages of the dentate population^2,29^. In recent decades, cross-sectional and longitudinal epidemiological studies on periodontitis in adults were performed in Chile^12^ and Brazil^14^. Moreover, analytical approaches designed to identify associated factors that could be risk indicators for gingival inflammation are nonexistent. Our main objective in this multi-center, population-based, cross-sectional and epidemiological study is to investigate the prevalence, severity, and risk indicators for gingival inflammation in representative samples of the adult populations of Porto Alegre (Brazil), Tucumán (Argentina), and Santiago (Chile).

## MATERIAL AND METHODS

### Study design, sampling, and sample sizes

The present cross-sectional, representative study utilized stratified, multistage probability samples of the civilian, noninstitutionalized adult populations in three South American cities. Data were collected between January and July of 2014.

Our sampling approach considered various age subgroups (18–19; 20–29; 30–39; 40–49; and ≥50 years of age). Considering previously published information that estimated a prevalence of gingivitis of 93.9%^21^ (average Gingival Index ≥0.5) with a precision rate of 95% and a 2% error, we determined that a sample size of 550 adults would be appropriate for each of the three cities in the study. To do so, the formula to estimate the prevalence of a population (n=Z^2^_1-α/2_ P(1-P)/e^2^) was used.

The study participants were selected using a multi-staged probability sampling process. Age groups were formed according to a proportional approach to the base population registries in the total of urban administrative regions in Porto Alegre, Tucumán, and Santiago, according to the last census in each city, and considering differences in gender and age.

The sampling process consisted of four stages: City (primary sampling units - 1^st^ stage); Tract census (2^nd^ stage); Blocks (3^rd^ stage); and Individuals within the age group (4^th^ stage). The cities were chosen according to logistics and interests in the three countries. Using maps of each city, primary census sectors were randomly chosen. The number of sectors in each city was determined according to the city size and census distribution. If the access to a primary census sector was not possible, the next available census sector was chosen. In each census sector, the blocks were randomly chosen. On each block, households were consecutively approached according to the sector starting point, until the number of participants expected for each sector was reached. Places such as nursing homes and commercial establishments were not included. When no potential participants were available for examination in a household, the next household was visited.

Candidates who have expressed an interest in participating in the study were selected based on the following criteria: 18 years of age or older, healthy, and with at least four permanent teeth. Were excluded from the study candidates needing antibiotic prophylaxis prior to dental examination, women who were pregnant or breastfeeding, individuals with fixed orthodontic appliances, or individuals who chronically used nifedipine, cyclosporine, phenytoin, or any prescription medicines that might interfere with the study outcome.

The protocol used for this study is in accordance with the Declaration of Helsinki and was reviewed and approved by the Institutional Review Boards of the University of Chile, Federal University of Rio Grande do Sul, and Maimonides University. All study participants were informed about the aims of the study and signed an informed consent form.

### Clinical evaluation and sociodemographic and behavioral data

A sociodemographic and general health interview was conducted and a structured questionnaire, consisting of open and closed questions about demographics, habits, attitudes, and knowledge related to oral health, was designed and administered to all participants. This questionnaire was tested at each of the three study sites and adapted according to the necessities of the local population. Finally, a complete dental examination was performed on all participants in the study.

Prior to the initiation of the study, the principal investigators and examiners met in Porto Alegre in order to standardize diagnostic criteria with the reference examiner (CR). Intra- and inter-examiner kappa coefficients for the Visible Plaque Index, Calculus Index, and Gingival Index were above 0.7. In addition, the structured questionnaire was standardized for each of the three study locations.

Each team consisted of one clinical examiner, totaling three dental examiners (RC, AT, FS) and each conducted exams using a manual periodontal probe (UNC-15) and mobile dental units. Good clinical practice standards were used and warranted. Periodontal clinical parameters were evaluated in all teeth, excluding third molars. The parameters evaluated were Visible Plaque Index (VPI), Calculus Index (CI), and Gingival Index (G-Index). Visual plaque assessment was determined using absence (0) or presence (1) of dental plaque according to Ainamo & Bay. The Löe modification of the Löe-Silness index was used to evaluate gingival health. Each tooth was divided into six surfaces, three facial and three lingual, as follows: 1) mesio-facial; 2) mid-facial; 3) disto-facial; 4) mesio-lingual; 5) mid-lingual; and 6) disto-lingual. Third molars and those teeth with cervical restorations or prosthetic crowns were excluded from the scoring procedure. Absence (0) or presence (1) of calculus was scored in lower anterior teeth (CI). Each tooth was divided into three lingual surfaces, as follows: disto-lingual, medio-lingual, and mesio-lingual. At the end of clinical examinations, those participants who were diagnosed with periodontal pathologic conditions were provided a written report of their condition and advised to seek an oral health consultation.

**Questionnaire:**
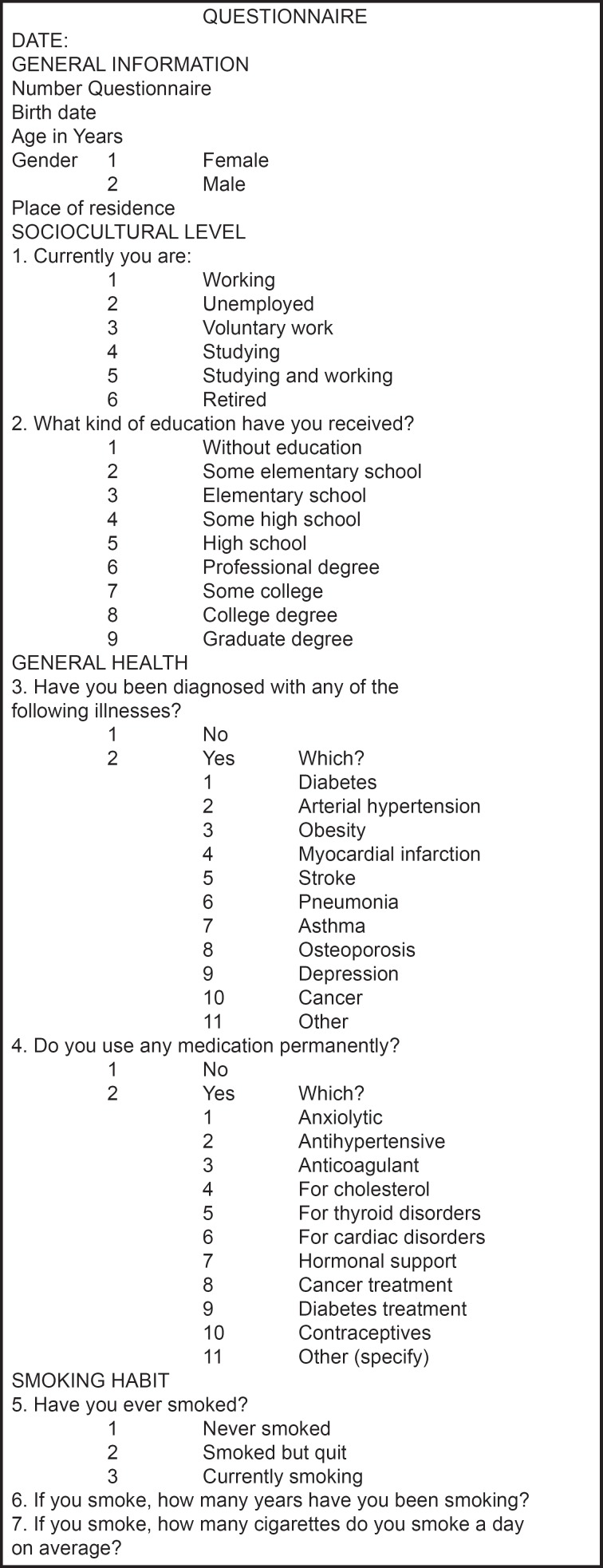
Structured questionnaire, consisting of open and closed questions about demographics, habits, attitudes, and knowledge related to oral health

### Definition of variables

The prevalence of gingival inflammation was defined as the percentage of study participants with a mean Gingival Index (G-Index) ≥0.5. Gingival Bleeding Index (GBI) was defined as the percentage of sites with G-Index ≥2. When appropriate, estimates were isolated for interproximal sites. Severity of gingival inflammation was defined as G-Index 0.5–1.0 for mild, 1.1–2.0 for moderate, and >2.0 for severe gingival inflammation. Study participants were classified by their smoking habits as either nonsmokers or smokers. Smokers were further classified as light (5 cigarettes *per* day), moderate (5–10 *per* day), and heavy (over 10 *per* day). The educational level of the study participants was also categorized as ≤12 or >12 years of school. The G-Index, VPI, GBI, and CI indices are represented in the data as mean values.

### Statistical analyses

Continuous data are presented as means ± SDs, and categorical variables are presented as percentages. The data are stratified according to sociodemographic, behavioral, and periodontal values. Chi-square tests were applied to compare distributions of periodontal variables between age groups and centers; to assess differences in the means and percentages, Mann-Whitney or Kruskall-Wallis tests were applied, and statistical analyses were performed using a statistical software package (Stata/IC 13.1). A multi-variable logistic regression model was built to assess the contribution of each variable (age, gender, smoking, and social or cultural factors). The occurrence of gingival inflammation was considered if G-Index ≥0.5. In addition, variables were analyzed in the model and allowed if the coefficient was modified by at least 10%. VPI was dichotomized at 30%. Odds ratios were calculated with 95% confidence intervals, and statistical significance was defined as p<0.05.

## RESULTS

A total of 1650 adults from Porto Alegre, Tucumán, and Santiago participated in this study. Females represent 52.5% of the overall study participants, while one third of the participants are in the >50 years age group.

### Prevalence of gingival inflammation

Table 1 shows that the overall prevalence of gingival inflammation (mean G-Index ≥0.5) for the study population is 95.6%. The Santiago and Porto Alegre participants present a significantly higher prevalence of gingival inflammation (p<0.001) at 99.1% and 97.3%, respectively, as compared with participants from Tucumán, at 90.4%. Only in Porto Alegre the prevalence of gingival inflammation in the ≥50 years age group is significantly higher than in the 40–49 and 20–29 years age groups (p<0.05). When the total sample is considered, a significant difference is observed between the prevalence of gingival inflammation in the 20–29 (92.3%) and the ≥50 years age groups (97.3%, p<0.05) for Porto Alegre and Tucumán, but not for Santiago.

**Table 1:** Prevalence of gingival inflammation (mean G-lndex≥0.5) according to age *per* city

Age (years)	PORTO ALEGRE**		TUCUMÁN		SANTIAGO		TOTAL**			
	Gingival Inflammation		Gingival Inflammation		Gingival Inflammation		Gingival Inflammation		Total	
	n	(%)	n	(%)	n	(%)	n	(%)	n	(%)
18–19	24	100	29	93.5	24	100	77	97.5	79	4.8
20–29	98	94.2	125	85.0	126	99.2	349	92.3	378	22.9
30–39	115	96.6	115	91.3	101	98.1	331	95.1	348	21.1
40–49	102	95.3	86	94.5	102	100	290	96.7	300	18.2
>50	196	100	142	91.6	192	99.0	530	97.3	545	33.0
Total	535	97.3*	497	90.4*	545	99.1*	1 577	95.6	1 650	100

* p-value <0.001, Fisher's exact test to assess differences between prevalence of gingival inflammation Tucumán versus P. Alegre and Santiago

** p-value <0.05, Fisher's exact test to assess differences between prevalence of gingival inflammation and age

Considering the total sample of the present study, no statistically significant difference is observed between genders (Table 2). In the entire sample, individuals with ≤12 years of education present a gingival inflammation prevalence that is higher than those with >12 years of education (prevalence of 97.3% versus 92.2%, respectively, p<0.0001), but this difference is most pronounced and significant only for the sample of Tucumán (94.9% versus 81.0%, p<0.05) when the three city samples are separately analyzed.

**Table 2:** Prevalence of gingival inflammation according to risk indicators per city

Variables		PREVALENCE OF GINGIVAL INFLAMMATION BY CITY AND TOTAL										
		n	(%)	PORTO ALEGRE		TUCUMÁN		SANTIAGO		TOTAL		p-value**
				n	(%)	n	(%)	n	(%)	n	(%)	
Gender	Female	867	52.6	275	98	274	92.6	286	98.3	835	96.3	
	Male	783	47.5	260	96.3	223	87.8	259	100	742	94.8	0.127
Education (years)	≤12	1 099	66.6	424	97.9	352	94.9*	293	99.3	1 069	97.3*	
	>12	551	33.4	111	94.9	145	81.0	252	98.8	508	92.2	0.000
Smoking	No	1 180	71.5	384	96.7	368	89.5	368	98.9	1 120	94.2	
	Current (light)	171	10.4	17	100.0	57	96.6	94	98.9	168	98.3	
	Current (moderate)	76	4.6	24	96.0	17	94.4	33	100.0	74	97.4	
	Current (heavy)	223	13.5	110	99.1	55	88.7	50	100.0	215	96.4	0.194
Self-reported diabetes	No diabetes	1 528	92.6	489	97.0	493	90.3	473	99.0	1 455	95.2	
	Yes	122	7.4	46	100	4	100	72	100	122	100*	0.005
Self-reported hypertension	No hypertension	1 332	80.7	379	96.4	443	89.5	441	99.3	1 263	94.8	
	Yes	318	19.3	156	99.4	54	98.2*	104	98.1	314	98.7*	0.001
Plaque Index	<30%	58	3.5	11	64.7	7	21.2	6	75.0	24	41.4	
	≥30%	1 592	96.5	524	98.3*	490	94.8*	539	99.5*	1 553	97.6*	0.000

* p-value <0.05, Fisher's exact test, to assess differences between prevalence of gingival inflammation for each variable and city

** p-value, Fisher's exact test, to assess differences between prevalence of gingival inflammation in the total sample for each variable

Participants who smoke present a higher prevalence of gingival inflammation as compared with nonsmokers, but the difference was not statistically significant (p=0.194). Those participants with self-reported diabetes and hypertension also present a significantly higher prevalence of gingival inflammation as compared with those not reporting these conditions. Within cities, only in Tucumán there is a statistically significant difference in gingival inflammation prevalence between those who self-report hypertension and those who do not (98.2% versus 89.5%, respectively, p<0.05). Adults with a visible plaque index ≥30% present a significantly higher prevalence of gingival inflammation than those with visible plaque index <30% (97.6% versus 41.4%, respectively, p<0.0001) (Table 2).

Table 3 describes all clinical variables examined in this study (mean G-Index, mean GBI, mean VPI, and mean CI). Regarding all of these variables, higher values are observed in individuals with higher gingival inflammation prevalence, compared with lower values in healthy adults (p<0.001) in the three cities. In participants with gingival inflammation, the mean G-Index in interproximal sites is 1.35 (±0.43) and in all sites is 1.33 (±0.43), without statistically significant differences. Study participants from Santiago had the highest average gingival index (1.73) compared with those from Tucumán and Porto Alegre, at 1.11 and 1.12, respectively (p<0.001). The mean VPI in interproximal sites is higher than the mean plaque index for all sites examined (81% versus 75%, respectively) for the total study population. Significant differences for the city of Porto Alegre and Santiago are found between interproximal VPI and total VPI. The rates of interproximal VPI in individuals with gingival inflammation in Santiago, Porto Alegre, and Tucumán are 89%, 80%, and 74%, respectively, while the mean interproximal VPI values in healthy adults are 45%, 30%, and 52%, for Santiago, Tucumán, and Porto Alegre, respectively, with significant differences between them (p<0.001) (Table 3). The mean CI rates in individuals with gingival inflammation are 89% in Porto Alegre, 74% in Tucumán, and 81% in Santiago with significant differences between them, while the CI rates in healthy adults are 52%, 26%, and 41%, respectively (Table 3). GBI is higher in individuals classified as having gingival inflammation (41%) as compared with healthy adults (2%, p=0.001), and GBI is higher in Santiago as compared with Porto Alegre and Tucumán (Table 3).

**Table 3:** Average G-Index, Interproximal Inter G-Index, Gingival Bleeding Index (GBI), Visual Plaque Index (VPI), Interproximal VPI, and Calculus Index (CI)

	PORTO ALEGRE				TUCUMÁN SANTIAGO								TOTAL				p-value*	p-value**	p-value***	p-value****
	(n = 550)				(n = 550)				(n = 550)				(n = 1650)							
Variable	Healthy		GI		Healthy		GI		Healthy		GI		Healthy		GI					
	n=15		n=535		n=53		n=497		n=5		n=545		n=73		n=1577					
	mean	SD	mean	SD	mean	SD	mean	SD	mean	SD	mean	SD	mean	SD	mean	SD				
G-Index	0.44	0.05	1.12	0.34	0.36	0.11	1.11	0.35	0.24	23.4	1.73	0.28	0.37	0.12	1.33	0.44	0.000	0.424	0.000	0.000
Inter G-Index	0.49	0.08	1.17	0.32	0.36	0.11	1.09	0.35	0.30	0.28	1.76	0.28	0.38	0.13	1.35	0.43	0.000	0.002	0.000	0.000
GBI	3.1	3.0	22.9	18.4	0.02	2.3	23.0	19.7	2.2	2.2	74.1	23.4	2.3	2.5	40.6	31.9	0.000	0.408	0.000	0.000
VPI	35.8	18.8	70.5	18.1	29.4	12.1	73.5	20.0	44.2	21.9	81.6	18.1	31.7	14.8	75.2	19.4	0.001	0.001	0.000	0.000
Inter VPI	45.1	22.5	79.0	17.1	30.0	12.5	74.0	20.3	52.4	27.1	88.9	16.0	34.6	17.7	81.2	18.8	0.004	0.002	0.000	0.000
CI	52.0	34.9	88.5	21.1	25.9	26.9	73.8	32.0	41.2	28.5	81.1	27.2	32.0	30.2	81.3	27.7	0.003	0.000	0.000	0.003

G-Index, Gingival Index; Inter G-Index, Interproximal Gingival Index; GBI, Gingival Bleeding Index; VPI, Visual Plaque Index, Inter VPI, Interproximal Visual Plaque Index; CI, Calculus Index. Mann-Whitney test or Kruskall-Wallis adjusted p-value for significance, as a appropriate, to assess; differences between healthy versus gingival inflammation for all cities (*), prevalence of gingival inflammation Porto Alegre versus Tucumán (**), prevalence of gingival inflammation Porto Alegre versus Santiago (***) and prevalence of gingival inflammation Tucumán versus Santiago (****)

### Severity of gingival inflammation

Tables 4a and 4b present mean G-Index as restricted to interproximal surfaces as well as mean GBI according to age and city. GBI means of 32.5%, 37.7%, 37.4%, 41.6%, and 45.2% are found for 18–19 year olds, 20–29 year olds, 30–39 year olds, 40–49 year olds, and ≥50 year olds, respectively (Table 4a). Participants from Santiago showed the highest mean GBI (Table 4a). Regarding the severity of gingival inflammation, 22.5% of participants examined have mild gingival inflammation, 74.0% have moderate gingival inflammation, and 3.6% have severe gingival inflammation. In Santiago, the vast majority of adults present moderate gingival inflammation (90.6%) and, for all age groups, the gingival inflammation prevalence is higher than what is observed in Porto Alegre and Tucumán (Table 4b).

**Table 4a:** Average Gingival Index (total and interproximal) and average Gingival Bleeding Index by age and city

		Gingival Index					Interproximal Gingival Index					Gingival Bleeding Index				
	Age (years)	Mean	IC 95%	p-value *	p-value **	p-value ***	mean	IC 95%	p-value *	p-value **	p-value ***	mean	IC 95%	p-value *	p-value **	p-value ***
PORTO ALEGRE	18–19	1.00	0.89,1.11	0.005			1.06	0.95,1.16	0.010			18.5	1.0,56.0	0.344		
	20–29	1.07	1.01,1.13				1.12	1.07,1.18				21.1	0.0,72.0			
	30–39	1.07	1.02,1.13				1.13	1.07,1.18				20.6	1.0,78.0			
	40–49	1.11	1.05,1.18				1.16	1.10,1.22				22.3	1.0,94.0			
	≥50	1.19	1.14,1.25				1.23	1.18,1.28				25.9	0.0,100			
TUCUMÁN	18–19	1.15	1.00,1.29	0.188	0.683		1.12	0.97,1.27	0.229	2.263		23.6	15.9,31.3	0.285	1.358	
	20–29	1.07	1.01,1.13		2.535		1.05	1.00,1.11		0.078		21.0	18.0,24.1		2.333	
	30–39	1.10	1.04,1.16		1.175		1.07	1.01,1.13		0.556		21.9	18.8,25.0		1.415	
	40–49	1.08	1.01,1.16		1.043		1.06	0.99,1.14		0.042		21.6	17.0,26.2		0.422	
	≥50	1.17	1.11,1.23		2.167		1.15	1.08,1.21		0.093		26.4	22.7,30.1		2.969	
SANTIAGO	18–19	1.51	1.34,1.67	0.000	0.000	0.005	1.55	1.39,1.70	0.000	0.000	0.000	57.1	44.8,69.4	0.000	0.000	0.000
	20–29	1.66	1.61,1.70		0.000	0.000	1.68	1.64,1.73		0.000	0.000	67.1	63.0,71.2		0.000	0.000
	30–39	1.72	1.66,1.79		0.000	0.000	1.76	1.70,1.82		0.000	0.000	74.2	69.3,79.1		0.000	0.000
	40–49	1.77	1.71,1.82		0.000	0.000	1.8	1.75,1.86		0.000	0.000	77.7	73.3,82.2		0.000	0.000
	≥50	1.78	1.74,1.81		0.000	0.000	1.8	1.77,1.84		0.000	0.000	78.8	75.8,81.7		0.000	0.000
TOTAL	18–19	1.21	1.12,1.31	0.000			1.23	1.14,1.32	0.000			32.5	26.2,38.7	0.002		
	20–29	1.28	1.24,1.33				1.3	1.26,1.34				37.7	34.6,40.8			
	30–39	1.28	1.23,1.33				1.3	1.25,1.35				37.4	34.0,40.8			
	40–49	1.33	1.28,1.39				1.36	1.30,1.41				41.6	37.7,45.5			
	>50	1.40	1.36,1.44				1.42	1.38,1.45				45.2	42.4,48.0			

Kruskal-Wallis adjusted p-value for significance, to assess differences between ages versus each Index by city and total (p-value*), differences between Porto Alegre versus Tucumán or Santiago in each Index (p-value**).

differences between Tucumán versus Santiago in each Index (p-value***)

**Table 4b:** Severity (mild, moderate, and severe) of gingival inflammation (Gl) per age and city

			MILD GI			MODERATE GI			SEVERE GI				
	Age (years)	n	%	IC 95%	n	%	IC 95%	n	%	IC 95%	p-value*	p-value**
PORTO ALEGRE	18–19	13	54.2	34.2,72.9	11	45.8	27.1,65.8	0	0.0			
	20–29	37	37.8	28.7,47.8	61	62.2	52.2,71.3	0	0.0			
	30–39	54	47.0	38.0,56.1	57	49.6	40.5,58.7	4	3.5	1.3,9.0	0.090	
	40–49	41	40.2	31.1,50.0	58	56.9	47.0,66.2	3	2.9	0.9,8.8		
	≥50	64	32.7	26.4,39.6	126	64.3	57.3,70.7	6	3.1	1.4,6.7		
TUCUMÁN	18–19	12	41.4	24.9,60.0	16	55.2	36.8,72.2	1	3.5	0.4,21.5		
	20–29	55	44.0	35.5,52.9	69	55.2	46.4,63.7	1	0.8	0.1,5.5		
	30–39	48	41.7	33.0,51.0	65	56.5	47.3,65.3	2	1.7	0.4,6.7	0.300	
	40–49	42	48.8	38.4,59.4	41	47.8	37.3,58.3	3	3.5	1.1,10.4		
	≥50	51	35.9	28.4,44.2	90	63.4	55.1,70.9	1	0.7	0.1,4.9		
SANTIAGO	18–19	3	12.5	4.0,33.0	21	87.5	67.0,96.0	0	0.0			0.007
	20–29	2	1.6	0.4,6.2	118	93.7	87.8,96.8	6	4.8	2.1,10.2		0.000
	30–39	6	6.0	2.7,12.7	90	89.1	81.3,93.9	5	5.0	2.1,11.4	0.230	0.000
	40–49	3	2.9	0.9,8.8	93	91.2	83.8,95.4	6	5.9	2.7,12.5		0.000
	≥50	4	2.1	0.8,5.4	176	91.7	86.8,94.8	12	6.3	3.6,10.7		0.000
TOTAL	18–19	28	36.4	26.4,47.7	48	62.3	51.0,72,5	1	1.3	0.2,8.7		
	20–29	94	26.9	22.5,31.8	248	71.1	66.1,75.6	7	2.0	1.0,4.2		
	30–39	108	32.6	27.8,37.9	212	64.1	58.7,69.1	11	3.3	1.9,5.9	0.019	
	40–49	86	29.7	24.7,35.2	192	66.2	60.6,71.4	12	4.1	2.4,7.2		
	≥50	119	22.5	19.1,26.2	392	74	70.0,77.5	19	3.6	2.3,5.6		

Chi-square test, to assess differences between ages versus severity for each city and total (p-value*), differences between cities by each group (p-value**)

### Risk indicators of gingival inflammation

The multivariate logistic regression model, which was designed to assess indicators that could be related to gingival inflammation prevalence (G-Index≥0.5) in the adult samples, demonstrates that subjects with higher CI mean (OR=18.59); with a VPI ≥30% (OR= 14.56); living in Santiago (OR=7.17); having ≤12 years of schooling (OR=2.18), and females (OR=1.93) are more likely to present gingival inflammation. This model was adjusted for age, presence of diabetes, and self-reported hypertension and smoking (Table 5).

**Table 5:** Multivariate analyses of factors associated with gingival inflammation (average G-Index ≥0.5). The variables that are statistically for any city or to the total are shown

		TOTAL (n=1650)			PORTO ALEGRE (n=550)			TUCUMÁN (n=550)			SANTIAGO (n=550)		
Variables		OR	CI 95%	p-value	OR	CI 95%	p-value	OR	CI 95%	p-value	OR	CI 95%	p-value
Gender	Male												
	Female	1.93	1.03,3.59	0.039	6.48	1.32,31.86	0.021	1.59	0.72,3.53	0.254	0.00	0.00	0.997
Education	>12												
(years)	≤12	2.18	1.19,4.02	0.012	1.29	0.33,5.06	0.713	3.21	1.46,7.08	0.004	1.88	0.24,15.06	0.550
CI (mean)		18.59	7.13,48.50	0.000	25.66	3.89,169.10	0.001	18.24	5.44,61.19	0.000	36.61		0.099
												0.51,2634.86	
VPI	<30%												
	≥30%	14.56	6.75,31.40	0.000	10.78	2.06,56.49	0.005	21.10	7.33,60.71	0.000	18.70	1.30,269.52	0.031
City	Tucumán												
	Porto Alegre	1.75	0.81,3.79	0.153									
	Santiago	7.17	2.58,19.93	0.000									

Logistic regression model for the presence of gingival inflammation (average G-index ≥0.5) adjusted for gender, age, educational level, self-reported diabetes, self-reported hypertension, smoking, visual plaque index, calculus index for each city and for the total sample by city. The variables that are statistically significant for any city or to the total are shown

## DISCUSSION

The population examined in the present study is comprised of a random sample of individuals aging 18 years or older from Porto Alegre (Brazil), Tucumán (Argentina), and Santiago (Chile). To our knowledge, this is the first study conducted to assess the prevalence of gingival inflammation in a representative sample of adult populations from three Latin American cities. The sampling strategy that we employed was successful in achieving a representative and balanced sample of participants, since the individuals examined in each age group for each of the three cities are in the same proportion as in the whole study population for the three cities combined. In this study, 95.6% of the 1650 adults examined from three South American cities present a G-Index >0.5. This information corroborates the data reported for adults from Jordan, China, and the United States of America, which demonstrated that adults with a G-Index >0.5 comprise 75.8%, 97.9%, and 93.9% of their respective populations^1,21,30^. Comparing our results with that from previous studies is somewhat hindered by the use of different nomenclature and diagnostic criteria across studies. The Community Periodontal Index (CPI) was used to report the occurrence of probing pocket depth, calculus, and gingival inflammation in the Hungarian adult population, and gingival bleeding (CPI=1) was observed in 8% of the population^15^. The National Health and Nutrition Examination Survey III (NHANES III) conducted in the USA between 1988–1994 demonstrated that 50% of the adult USA population had gingival inflammation, using gingival bleeding as the criterion^3^. A study conducted in Italy, using bleeding on probing (BoP) as the criterion, determined that the prevalence of individuals showing at least one site positive for BoP was 99%^11^. All the aforementioned studies demonstrate that the occurrence of gingival inflammation is almost universally correlated with poor gingival conditions. In addition, there is a large discussion in the literature regarding how to define disease criteria. It would be interesting to develop guidelines to suggest which criteria should be standardized for use in epidemiological studies for gingival conditions^16^. Concerning the severity of gingival inflammation, the present study shows that 22.5% of adults have mild gingival inflammation, 74.0% have moderate gingival inflammation, and 3.6% have severe gingival inflammation. In contrast, representative studies conducted in Europe and Australia demonstrated that the prevalence of severe gingival inflammation is comparably much higher (17% and 19.7%, respectively) in these populations^15,17^. The multivariate logistic regression models that we employed show that a female with ≤12 years of schooling, a higher dental calculus index, ≥30% of VPI, and living in Santiago has an increased likelihood of presenting gingival inflammation, when adjusting for age, presence of diabetes, and self-reported hypertension and smoking. In this study, to live in Santiago was a risk indicator for presenting GI. It could be because these subjects had lower oral hygiene habits compared with those from the other cities. Indeed, subjects with presence of GI belonging to Santiago had a higher average percentage of VPI, compared with subjects with GI of Tucumán and Porto Alegre. In fact, the plaque index has been associated with increased presence of gingival inflammation and worst level of oral hygiene in other populations^9^. There are limited studies at the Latin American level on the prevalence of periodontal conditions^27^, which allow contrast with the findings in this study. Although subjects belonging to Santiago present greater gingival inflammation and increased risk of it, is based with those reported for that country, where Chilean adults had a high prevalence of periodontal destruction and indicators of gingival inflammation^12^.

The present study also demonstrates a socioeconomic gradient in the oral health of the populations examined. Participants from lower socioeconomic groups present significantly poorer gingival health as compared with participants in higher socioeconomic groups^4^. In this regard, different explanations have been raised in the literature to explain such findings. For example, those in lower socioeconomic situations are also more likely to smoke, and smoking has been found as a significant risk factor for periodontitis^10^. On the other hand, individuals with higher socioeconomic status are likely to have more positive attitudes regarding oral hygiene and self-care, and better access to available health care options. Therefore, low incomes and low levels of education seem to be variables with good predictability for periodontal diseases^7^. The prevalence of gingival inflammation in females found in the present study contradicts results from Greece, where women have been shown to have better oral hygiene and gingival status than men^22^. Perhaps this difference is more about culture than gender. It should be noted that only when adjusting the multivariate model by confounding variables, female gender appears as a possible risk indicator for the presence of gingival inflammation. On the other hand, the longitudinal recovery of bleeding sites was positively influenced in females^25^.

Regarding the self-reported diabetes and hypertension among our participants, 7.4% have diabetes and 19.3% have hypertension. Participants with self-reported diabetes and hypertension present a significantly higher prevalence of gingival inflammation than those not reporting these conditions. Previous studies have reported comparable results regarding this association between type 2 diabetes and periodontal disease^20^.

We find that current smokers comprise 28.8% of our total study participants, and these smokers present a slightly higher prevalence of gingival inflammation than nonsmokers in our study, but this finding was not statistically significant. These results contrast with the findings of Muller, et al.^25^ (2002), in which study participants (soldiers of the German Armed Forces serving between December 1999 and May 2000) who smoked were found to have more prevalent bleeding on probing and more calculus than nonsmokers. Smoking was first identified as a risk factor in periodontal diseases from an analysis of data by Ismail, et al.^18^ from data collected from 1971 to 1975. An analysis by the National Health and Nutrition Examination Survey in the U.S. (NHANES I) was able to demonstrate an association between smoking and periodontal diseases that was independent of oral hygiene, age, or other probable risk factors. Since then, there has been enough evidence to identify smoking as a risk factor for periodontitis^13^. Studies performed in randomly chosen groups of patients demonstrated that tobacco use and oral hygiene are risk indicators for periodontitis; in particular, smokers were invariably shown to have a higher prevalence and progression of destructive periodontal diseases^24^.

There is a strong need in Latin America to focus on more effective intervention programs to prevent and control periodontal diseases at national levels. It should be emphasized that since periodontitis begins as gingivitis, it is reasonable to conclude that the control of gingival inflammation can be beneficial to the population as a whole to prevent both the onset and the progression of periodontal damage caused by periodontitis. While the disability-adjusted life-years (DALYs) due to severe periodontitis and untreated caries have increased since 1990, those due to severe tooth loss have decreased. Oral conditions are all ranked among the top 100 detailed causes of DALYs^23^. These findings highlight the challenge in responding to the diversity of urgent oral health needs worldwide, particularly in developing communities.

Gingival inflammation, which could be considered a reversible and easily controlled disease in stage of gingivitis, is found to be highly prevalent among adult study participants from the three cities in Latin America of this study. In addition, these individuals are also more likely to attend preventive or follow-up visits because socioeconomic characteristics (such as income and level of education) influence the pattern and type of dental services used^6^. Poor awareness of the importance of periodontal health and the consequences of the disease among the public and even among some general dental practitioners is one of the most common reasons for failure to control and treat periodontal diseases effectively on a population basis^19^. We recommend that effective intervention programs for the prevention and control of periodontal diseases should be implemented at national levels, and the need for such implementation seems to be extremely important in the three countries we studied here. We believe that there is a strong need across Latin America to improve the population's self-awareness about oral health through better oral health education that promotes good oral hygiene and regular dental care.

This study was aimed to detect gingival inflammation instead of establishing the diagnosis of either gingivitis or periodontitis in order to determine the risk of gingivitis onset or periodontitis progression in the study population.

## CONCLUSIONS

Considering our findings together, it is possible to conclude that gingival inflammation is highly prevalent in the three Latin American cities studied. Overall, 95.6% of the participants aging 18 years or older had gingival inflammation, considering that more than two-thirds have moderate gingival inflammation and 3.6% have severe gingival inflammation. In general terms, the presence of gingival inflammation is positively associated with risk indicators such as gender, socioeconomic variables, and the presence of plaque. The present investigation serves as the basis for a longitudinal analysis of oral health in populations of South American adults, and for the development of strategies to improve the health care systems that serve them.
